# Modulation of the Gut Microbiota by *Nopalea cochenillifera* (Prickly Pear Cactus) Contributes to Improved Lipid Metabolism and Immune Function

**DOI:** 10.3390/nu17172844

**Published:** 2025-08-31

**Authors:** Sayaka Yokoyama, Amane Kikuchi, Hideaki Takahashi, Hinako Ushimaru, Hibiki Yamaguchi, Chikako Yamada, Kotoyo Fujiki, Hana Kozai, Suzuno Ota, Tadashi Fujii, Yoshiki Hirooka, Takumi Tochio, Mamoru Tanaka

**Affiliations:** 1 Graduate School of Bioscience and Biotechnology, Chubu University, 1200 Matsumoto, Kasugai 487-8501, Japan; yokoyama-s@kinjo-u.ac.jp (S.Y.); gr24011-7447@sti.chubu.ac.jp (A.K.); gr25028-0404@sti.chubu.ac.jp (H.Y.); hkozai@fsc.chubu.ac.jp (H.K.); gr25802-9474@sti.chubu.ac.jp (S.O.); 2 Department of Food and Nutritional Environment, Kinjo Gakuin University, 2-1723 Omori, Moriyama-ku, Nagoya 463-8521, Japan; ushimaru@kinjo-u.ac.jp; 3 Graduate School of Nutritional Sciences, Nagoya University of Arts and Sciences, Nisshin 470-0196, Japan; h-takahashi@biosislab.co.jp (H.T.); yamada-c@nuas.ac.jp (C.Y.); kfujiki@nuas.ac.jp (K.F.); 4 Department of Gastroenterology and Hepatology, Fujita Health University, Toyoake 470-1192, Japan; tadashi.fujii@fujita-hu.ac.jp (T.F.); yoshiki.hirooka@fujita-hu.ac.jp (Y.H.); t-tochio@fujita-hu.ac.jp (T.T.); 5 BIOSIS Lab, Co., Ltd., Toyoake 470-1192, Japan; 6 Department of Medical Research on Prebiotics and Probiotics, Fujita Health University, Toyoake 470-1192, Japan

**Keywords:** *Nopalea cochenillifera*, cholesterol, *Lachnospiraceae*, intestinal barrier function

## Abstract

**Background/Objectives**: *Nopalea cochenillifera (L.) Salm-Dyck* cladodes are rich in dietary fiber, polyphenols, and minerals, which are known to exert antioxidant and immunomodulatory effects. However, the mechanisms and active constituents have not been fully elucidated. In this study, we investigated the effects of continuous *N*. *cochenillifera* consumption on lipid metabolism, immune function, and the gut microbiota in mice. **Methods:** The feed was made using freeze-dried and powdered cladodes of *N. cochenillifera*. Male C57BL/6J mice were assigned to four groups: control diet (C), control diet plus 10% *N. cochenillifera* (CN), high-fat diet (FC), and high-fat diet plus 10% *N. cochenillifera* (FN). **Results:** Cactus supplementation reduced the body and liver weights that were elevated by the high-fat diet. Serum total cholesterol and free fatty acids were increased in the FC group compared with the C group, while cactus intake lowered these levels and enhanced fecal cholesterol excretion. Cactus consumption also elevated fecal total IgA and mucin contents. IL-4 expression in Peyer’s patches was significantly increased in the FN group compared with the FC group. Gut microbiota analysis showed significant differences in β-diversity, along with increased α-diversity and higher abundance of *Lachnospiraceae,* following cactus intake. **Conclusions:** These findings suggest that *N. cochenillifera* intake increases gut microbiota diversity, which enhances intestinal barrier function and thereby contributes to improved lipid metabolism and immune regulation.

## 1. Introduction

In Japan, the increasing prevalence of obesity has become a significant public health concern, primarily due to changes in dietary habits influenced by Western food culture, as well as insufficient physical activity. According to the National Health and Nutrition Survey conducted in 2023, obesity is a major risk factor for lifestyle-related diseases such as diabetes, dyslipidemia, and hypertension in men [1]. Given that these diseases are among the leading causes of death in Japan, preventing obesity and related lifestyle diseases is of critical importance. 

The human gastrointestinal tract is estimated to harbor over 1000 bacterial species and approximately 100 trillion microbial cells. In recent years, the gut microbiota has garnered increasing attention for its critical role in health and disease, particularly with respect to dietary influences on microbial composition and function. Takahashi et al. demonstrated that dietary supplementation with oligosaccharides, such as 1-kestose, alone or with inulin, alleviates anaphylactic symptoms by reducing rectal-temperature decline. Moreover, it suppresses the elevation of serum antigen-specific IgE levels, thereby preventing food allergy [2]. Short-chain fatty acids (SCFAs), including acetate, propionate, and butyrate, are metabolites produced by commensal gut bacteria. They regulate host immunity by inducing T-cell differentiation and maintain intestinal homeostasis through modulation of cytokine production by Th1 cells [3]. Among these SCFAs, butyrate, in particular, plays a crucial role in maintaining gut homeostasis because of its potent immunomodulatory effects. Conversely, dysbiosis, which is an imbalance in the gut microbial community, has been associated with metabolic disturbances and aberrant immune responses. High-fat diets can compromise intestinal epithelial barrier integrity by disrupting tight junction proteins and promoting mucosal inflammation, which collectively lead to increased intestinal permeability [4,5]. This allows for the translocation of endotoxins such as lipopolysaccharide (LPS) into the systemic circulation, thereby triggering low-grade systemic inflammation [6]. Chronic inflammation associated with obesity may, in turn, disrupt lipid metabolism and energy homeostasis through microbiota-mediated mechanisms and is considered a contributing factor in the pathogenesis of lifestyle-related diseases.

The population of developing countries is projected to increase by approximately 30% from 2010 to 2050, and, as a result, global food demand is expected to rise by a factor of 1.7. Meanwhile, global warming has raised serious concerns regarding the stability of the food supply, including declines in crop quality and agricultural productivity. Prickly pear cactus (*Nopalea cochenillifera (L.) Salm-Dyck*), a cactus species known for its tolerance to extreme environmental conditions, such as high temperatures and drought, has attracted attention as a potential food resource for enhancing food self-sufficiency in arid and semi-arid regions. In 2017, the Food and Agriculture Organization (FAO) of the United Nations highlighted the potential of cactus species, including *N. cochenillifera*, as crops capable of contributing to the mitigation of global food insecurity, further underscoring their value as sustainable plant resources. The family *Cactaceae* comprises approximately 30 genera and over 1450 species, which are classified into four subfamilies: *Pereskioideae*, *Opuntioideae*, *Maihuenioideae*, and *Cactoideae*. The subfamily *Opuntioideae* contains approximately 20 genera and 300 species, several of which are edible and cultivated for food purposes in more than 30 countries worldwide. Elucidating the functional properties of *N. cochenillifera* and adding novel value is essential for enhancing its credibility as a food resource and for promoting its broader utilization. It has been reported that the stems of prickly pear cactus contain dietary fiber; polyphenols; and minerals, such as calcium [7]. These components have been reported to exert physiological effects on lipid metabolism [8,9], immune function [10,11,12], and the gut microbiota [8,9,13]. Although prickly pear cactus has been reported to have anti-obesity [14,15], anti-inflammatory [16], and immune-modulating [17] effects, research conducted on *N. cochenillifera* remains limited, and its bioactivities and underlying mechanisms have yet to be fully elucidated. Most prior studies focused on isolated functions, and few have comprehensively evaluated immune function, lipid metabolism, and gut microbiota in an integrated manner. Therefore, this study aims to address these gaps by simultaneously assessing these physiological systems. In addition, while a substantial body of research has been conducted regarding the larger prickly pear species *Opuntia ficus-indica*, studies focusing on the smaller species *N. cochenillifera* remain limited [18].

Previous studies have primarily focused on individual functional properties of *N. cochenillifera*, such as its anti-obesity and anti-inflammatory effects, while comprehensive evaluations of multiple physiological functions, including lipid metabolism, immune responses, and gut microbiota modulation, remain limited. Evaluating *N. cochenillifera* for its potential in preventing obesity and lifestyle-related diseases, as well as its utility as a sustainable food resource, is essential for scientific validation of its efficacy. The aim of the present study was to investigate the effects of *N. cochenillifera* consumption on lipid metabolism, immune function, and gut microbiota.

## 2. Materials and Methods

### 2.1. N. cochenillifera Samples

The cladodes of *Nopalea cochenillifera* used for the diet were obtained from Goto Cactus (Kasugai, Japan) as products commonly sold for edible use. Cladodes approximately 10–15 cm in length were used, and the cactus powder was prepared according to the method described by Kozai et al. [17]. After harvesting, the cladodes were immediately freeze-dried and then ground into powder using a blender.

### 2.2. Mice

Seven-week-old C57BL/6J male mice were obtained from Japan SLC (Shizuoka, Japan). This strain is commonly employed to establish diet-induced obesity models, as it shows metabolic changes similar to those observed in human obesity, making it suitable for obesity- and diabetes-related research. This study was approved by the Chubu University Animal Experiment Committee (Approval No. 202210019), and all experiments were conducted in accordance with Chubu University Animal Experiment Regulations. During the experimental period, the mice were kept at a room temperature of 24 ± 3 °C, with a humidity level of 55 ± 10% and a 12/12 h light/dark cycle (light period: 8:00 A.M.–8:00 P.M.). The mice were divided into four groups, ensuring that the average body weight was similar across the groups: control (C) (*n* = 10), control diet + 10% *N. cochenillifera* (CN) (*n* = 10), high-fat diet (F2WTD) (FC) (*n* = 10), and high-fat diet (F2WTD) diet + 10% *N. cochenillifera* (FN) (*n* = 10). The mice were housed five per cage and individual mice were considered the experimental unit for statistical analyses. At the time of group assignment, body weight was measured to ensure no significant differences among groups. Cage positions within the facility were not rotated.

The nutritional composition of the feed is shown in Table 1. The experimental diets were prepared according to the recommendations of the American Institute of Nutrition and were based on the AIN-93G rodent diet. The C group was fed the AIN-93G diet, the F group was fed the F2WTD diet, and the CN and FN groups were fed the same diets supplemented with 10% cactus powder by weight, for 12 weeks. Each feed was purchased from Oriental Yeast Co., Ltd. (Tokyo, Japan). During the experimental period, the mice were allowed ad libitum access to feed and tap water. Nutritional analysis of each diet was performed by the Japan Food Research Laboratories (Aichi, Japan).

### 2.3. Animal Protocol

The animals were dissected at the end of the experimental period, without prior fasting. On the day of sacrifice, the mice were anesthetized via inhalation of isoflurane. The liver, spleen, cecum, cecum contents, visceral fat (mesentery fat, epididymis fat, and perineal fat), and triceps surae muscles (soleus and gastrocnemius) were collected. After recording the tissue weights, the samples were stored at −80 °C. Blood was collected from the portal vein and centrifuged (4 °C, 1500× *g*, 15 min) to isolate the serum, which was stored at −20 °C.

### 2.4. Serum Biochemical Evaluation

Serum levels of blood glucose, triglycerides (TGs), total cholesterol, HDL cholesterol, free fatty acids (FFAs), albumin, aspartate aminotransferase (AST), alanine aminotransferase (ALT), insulin, leptin, and total IgM and IgG were measured. Serum glucose concentrations were measured using a LabAssay^TM^ Glucose kit (Fujifilm Wako Pure Chemical Co., Ltd., Osaka, Japan). TG concentrations were measured using a LabAssay Triglyceride kit, total cholesterol concentrations using a LabAssay Cholesterol kit, HDL-cholesterol concentrations using a LabAssay HDL-Cholesterol kit, and FFA concentrations using a LabAssay NEFA kit (all from Fujifilm Wako Pure Chemical Co., Ltd., Osaka, Japan). Serum AST and ALT concentration were measured using a Test Wako kit (Fujifilm Wako Pure Chemical Co., Ltd., Osaka, Japan). Serum albumin concentrations were determined using an LBIS^TM^ Mouse Albumin ELISA kit (RTU), and serum insulin concentrations were measured using an LBIS Mouse Insulin ELISA kit (RTU) (both from Fujifilm Wako Pure Chemical Co., Ltd., Osaka, Japan). Serum leptin levels were measured using a Mouse/Rat Leptin Assay kit (Morinaga Institute of Biochemistry, Inc., Kanagawa, Japan). Serum total IgM and IgG levels were measured using an Invitrogen^TM^ IgM Mouse Uncoated ELISA kit and an Invitrogen IgG Mouse Uncoated ELISA kit (Thermo Fisher Scientific, Waltham, MA, USA). All assays were performed according to the manufacturers’ instructions.

### 2.5. Fecal Weight, Cholesterol Excretion, Immunoglobulin A (IgA), and Mucin Analysis

For 3 days prior to each evaluation point, mice were individually housed in separate cages, and feces were collected and lyophilized. The weight of the freeze-dried and pulverized feces was measured and recorded as the dry weight. Fecal cholesterol was determined by extracting lipids using the Folch method [19] and measuring cholesterol levels with the same assay kit used for the serum biochemical evaluation. Fecal total IgA and mucin assays were performed as previously described [20]. Total IgA concentrations in feces were measured using an Invitrogen^TM^ IgA Mouse Uncoated ELISA kit (Thermo Fisher Scientific, Waltham MA, USA). Mucins were quantified by fluorometric assay of fecal mucin (Cosmo Bio Co., Ltd., Tokyo, Japan). All assays were performed according to the manufacturers’ instructions.

### 2.6. Cytokine Expression in Peyer’s Patches

RNA extraction, reverse transcription, and quantitative polymerase chain reaction (qPCR) were performed as previously described [2]. RNA was extracted from Peyer’s patches, ileum, and colon using a BioMasher^®^ II Micro Tissue Homogenizer (DWK Life Sciences, Millville, NJ, USA) and an RNeasy^®^ Mini Kit (Qiagen, Hilden, Germany). RNA concentrations were measured using a Qubit™ RNA (Broad-Range) Assay Kit (Invitrogen, Carlsbad, CA, USA). Reverse transcription was conducted using a High-Capacity RNA-to-cDNA kit (Thermo Fisher Scientific, Waltham, MA, USA). Reverse transcription qPCR was performed on a QuantStudio 3 Real-Time PCR System (Thermo Fisher Scientific, Carlsbad, MA, USA). Reaction mixtures for each cDNA and primer set were prepared using PowerTrack SYBR Green Master Mix (Thermo Fisher Scientific, Waltham, MA, USA) according to the manufacturer’s instructions. Specific primers were designed to target the interleukin (IL)-2, IL-4, IL-6, and IL-10 genes, with the 18S rRNA gene serving as an internal gene. The primer sequences are listed in Supplementary Table S1. The amplification protocol consisted of an initial hold at 95 °C for 2 min, followed by 40 cycles of 15 s at 95 °C and 30 s at 60 °C. Melting curve analysis was performed post-amplification to confirm the specificity of the PCR products. Gene expression levels were calculated using the ΔΔCt method.

### 2.7. Analysis of Gut Microbiota

The 16S rRNA gene next-generation sequencing (NGS) of cecal DNA samples was performed following a previously described protocol [21]. The sequencing data were processed, analyzed statistically, and graphed using the EzBioCloud 16S database and 16S microbiome pipeline provided by ChunLab Inc. [22]. To assess the richness and evenness of the microbial samples, we used Chao1 estimation and the Shannon diversity index, representing α-diversity. The Unifrac distance metric was used to estimate β-diversity, which indicates the overall phylogenetic distance among the groups, and principal coordinate analysis (PCoA) was used to visualize the diversity. To test the differences in α- and β-diversity between groups, we used the Wilcoxon rank-sum test and permutational multivariate analysis of variance with 9999 permutations, respectively. The linear discriminant analysis effect size (LEfSe) algorithm was used to identify potentially characteristic bacterial families distinguishing each group from the others. This identification was based on a linear discriminant analysis score > 4.0. Values of *p* < 0.05 were considered to indicate statistical significance.

### 2.8. Quantification of Short-Chain Fatty Acids in Cecal Samples

The carboxyl groups of SCFAs (acetate, propionate, and butyrate) and lactate in each cecum sample were derivatized with 2-nitrophenyl hydrazide using a Short- and Long-Chain Fatty Acid Analysis Kit (YMC, Kyoto, Japan), with the improved kit protocol described previously [2].

### 2.9. Statistical Analysis

Statistical analyses were performed using individual mice as the experimental unit (*n* = 10 per group), rather than cages. All statistical analyses were performed using GraphPad Prism version 10.2.3 (GraphPad Software, San Diego, CA, USA). Data are expressed as the mean ± standard error of the mean (SEM). Statistical analyses of microbiota were conducted either by the nonparametric Mann–Whitney U test or by the Kruskal–Wallis test, as appropriate. All other statistical analyses were performed using parametric two-way analysis of variance (ANOVA), followed by Tukey’s multiple comparisons test as a post hoc test. Statistical significance was set at *p* < 0.05.

## 3. Results

### 3.1. Feed Intake and Energy Intake for Each Group

The feed intake and energy intake for each group are shown in Table 2. Because the calculations were performed per cage, statistical significance could not be determined. Feed intake tended to be higher in the groups fed diets containing cactus powder, while no clear difference in energy intake was observed between the groups fed diets with and without cactus powder.

### 3.2. Body Weight and Tissue Weights

The body and tissue weights are shown in Table 3. There were no significant differences in spleen weight, visceral fat mass, or triceps muscle mass among the groups. The final body weight was significantly decreased in the FN group compared with the FC group. Similarly, liver weight was significantly lower in the FN group than in the FC group. The cecum content weight did not differ between the FC and FN groups; however, it was significantly higher in the CN group compared with the other groups.

### 3.3. Serum Biochemical Evaluation and Immune Function Assessment

**Figure 1:**
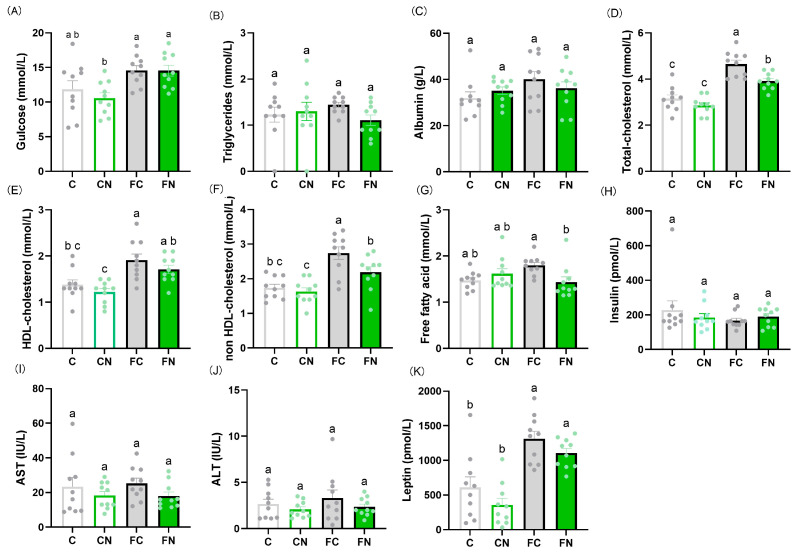
Effect of *N. cochenillifera* on serum levels of (**A**) glucose, (**B**) triglycerides, (**C**) albumin, (**D**) total cholesterol, (**E**) HDL cholesterol, (**F**) non-HDL cholesterol, (**G**) free fatty acids, (**H**) insulin, (**I**) AST, (**J**) ALT, and (**K**) leptin. Data are expressed as the mean ± SEM (*n* = 10). Means for variables without a common letter differ significantly (*p* < 0.05).

**Figure 2:**
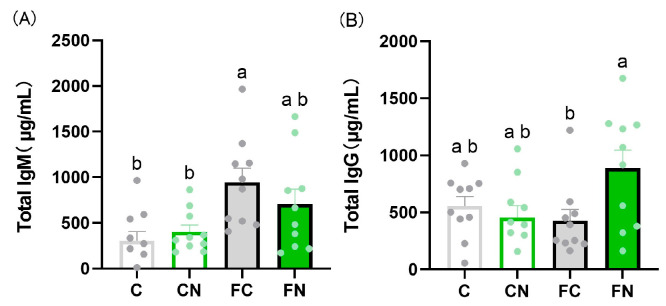
Effect of *N. cochenillifera* on (**A**) total IgM and (**B**) total IgG serum titers. Data are expressed as the mean ± SEM (*n* = 10). Means for variables without a common letter differ significantly (*p* < 0.05).

**Figure 3:**
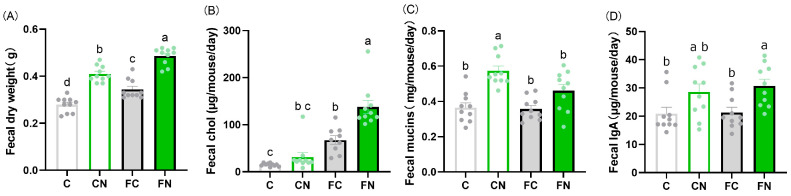
Effects of a diet containing *N. cochenillifera* on fecal weight, fecal mucin, and fecal IgA in mice. Feces collected 3 days prior to each evaluation point were freeze-dried and weighed. (**A**) Fecal dry weight. (**B**) Fecal cholesterol, (**C**) fecal mucin, and (**D**) fecal total IgA per fecal dry weight. Data are expressed as the mean ± SEM (*n* = 10). Means for variables without a common letter differ significantly (*p* < 0.05).

No significant differences were observed in blood glucose, serum triglycerides, albumin, insulin, AST, ALT, or leptin levels between groups with and without cactus supplementation (Figure 1A–C,H–K). By contrast, serum total cholesterol, HDL cholesterol, non-HDL cholesterol, and FFA levels were significantly lower in the FN group than in the FC group (Figure 1D–G). Serum IgM levels were elevated in the FC group (Figure 2A), while IgG levels were significantly higher in the FN group than in the FC group (Figure 2B). No significant differences were found in IL-6 or IL-10 expression in Peyer’s patches among the groups (Figure 3C,D). However, IL-4 expression was significantly increased in the FN group compared with the FC group (Figure 3B), whereas IL-2 expression tended to be decreased in the FN group compared with the FC group (*p* = 0.0788) (Figure 3A).

### 3.4. Fecal Weight and Measurement of Fecal Cholesterol, Total IgA, and Mucin

**Figure 4:**
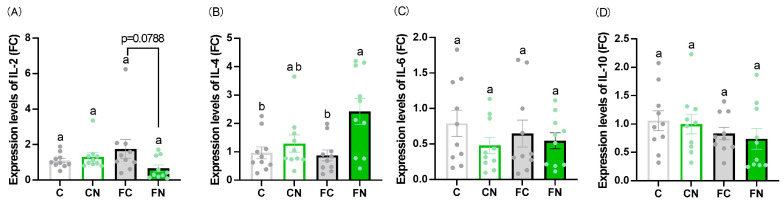
Effect of *N. cochenillifera* on cytokine production in Peyer’s patches. Gene expression levels of (**A**) IL-2, (**B**) IL-4, (**C**) IL-6, and (**D**) IL-10 in Peyer’s patches. Data are expressed as the mean ± SEM (*n* = 10). Means for variables without a common letter differ significantly (*p* < 0.05). FC: fold change.

Fecal weight was significantly increased in both the CN and FN groups (Figure 4A). Fecal cholesterol levels were significantly higher in the FN group than in the groups that received cactus supplementation (Figure 4B). Fecal mucin levels were significantly elevated in the CN group compared with the C group (Figure 4D). Fecal IgA levels were significantly increased in both the CN and FN groups compared with the C and FC groups, respectively (Figure 4A).

### 3.5. Analysis of the Intestinal Microflora in Cecum Contents

To investigate shifts in the microbial community resulting from cactus supplementation, cecal microbiota were characterized by 16S rRNA gene sequencing using next-generation sequencing (NGS). The obtained 16S rRNA sequencing data were deposited in the NCBI Sequence Read Archive (SRA) database under accession number PRJNA1287278.

**Figure 5:**
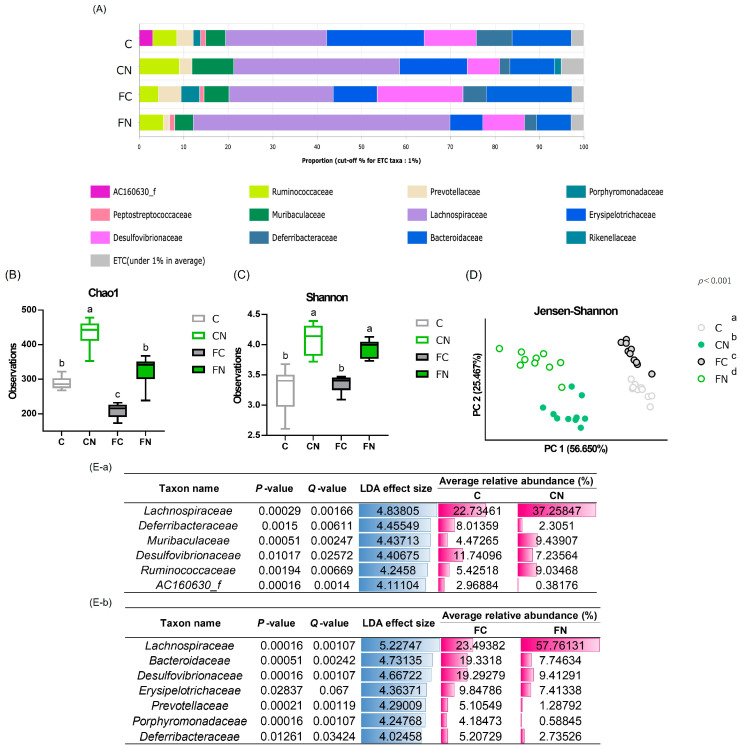
Effects of *N*. *cochenillifera* consumption on the intestinal microflora. (**A**) Average relative abundances (%) of cecal microbial taxa at the family level are shown across the four groups analyzed. Each bar represents the mean relative abundance of an identified bacterial species within each respective study group. (**B**) Chao1 index values. (**C**) Shannon index values. (**D**) Principal coordinate analysis based on weighted UniFrac distances. The first primary component is plotted on the horizontal axis (PC1), and the second primary component is plotted on the vertical axis (PC2). (**E**) Linear discriminant analysis (LDA) effect size was used to identify microbial taxa significantly associated with both cactus-fed groups compared with their respective control groups at the family level within the cecal microbiota. (**E**-**a**) Comparison between groups C and N. (**E**-**b**) Comparison between groups FC and FN. The AC160630_f family belongs to the order Bacteroidales. Means for variables without a common letter differ significantly (*p* < 0.05).

The microbial composition of the cecal microbiota was compared among the C, CN, FC, and FN groups. Figure 5A illustrates the fecal microbiota composition at the family level. The Chao1 index was significantly increased by cactus supplementation under both dietary conditions, while the Shannon diversity index was also significantly higher in the CN and FN groups than in C and FC groups, respectively (Figure 5B,C). β-diversity analysis revealed significantly different bacterial family distribution among the four groups (Figure 5D). LEfSe analysis at the family level revealed that cactus consumption significantly increased the abundance of *Lachnospiraceae* in both cactus-fed groups compared with their respective control groups (Figure 5E).

### 3.6. Measurement of Short-Chain Fatty Acids in Cecum Contents

**Figure 6:**
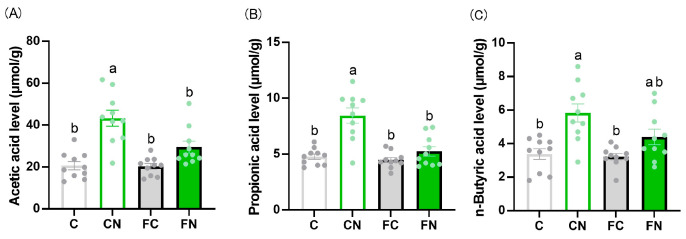
Effect of *N. cochenillifera* intake on short-chain fatty acid production. Changes in (**A**) acetate, (**B**) propionate, and (**C**) n-butyrate levels in cecal samples from mice fed *N. cochenillifera*. Data are expressed as the mean ± SEM (n = 10). Means for variables without a common letter differ significantly (*p* < 0.05).

The SCFAs in the cecal content samples were measured to evaluate the influence of cactus consumption on gut microbial metabolism. The levels of all analyzed SCFAs—acetic acid, butyric acid, and propionic acid—were significantly increased in the cactus-fed CN group compared with the control C group (Figure 6).

## 4. Discussion

The purpose of this study was to elucidate the effects of *N. cochenillifera* consumption on lipid metabolism, immune function, and the intestinal microbiota. Dietary supplementation of a high-fat diet with *N. cochenillifera* resulted in reduced serum total cholesterol levels, enhanced intestinal barrier integrity, increased abundance of butyrate-producing bacteria, and decreased levels of bacteria associated with dysregulated lipid metabolism. These findings suggest that *N. cochenillifera* intake may have a beneficial effect on lipid metabolism, immune responses, and the diversity of the gut microbiota.

A high-fat diet supplemented with *N. cochenillifera* suppressed body weight gain and increased the weights of cecal contents and feces. The dietary fiber content of the cactus powder used in this study was not directly analyzed. According to a previous report using the same preparation method [17], *N*. *cochenillifera* powder contains approximately 50% dietary fiber, of which about 80% is insoluble and 20% is soluble [17]. Thus, our findings may reflect increased dietary fiber intake, leading to reduced energy absorption efficiency and enhanced fecal bulk due to undigested polysaccharides and water-binding capacity. Such effects are consistent with previous studies on fermentable fibers, which have been shown to promote satiety, delay gastric emptying, and reduce overall energy intake [23,24]. In addition, serum biochemical analyses revealed reductions in total cholesterol and FFA levels. Elevated circulating FFAs exacerbate hepatic lipid accumulation [25], insulin resistance [26,27], and systemic inflammation [28]. Therefore, the observed reduction in FFA levels suggests that *N. cochenillifera* may help mitigate obesity-associated metabolic disturbances. Dietary fiber, particularly soluble fiber, is known to bind cholesterol and bile acids in the intestinal lumen, thereby inhibiting their absorption. Although insoluble fiber is less effective, it also contributes to lowering cholesterol levels [29]. On the basis of these mechanisms, we hypothesized that the cholesterol-lowering effect of *N. cochenillifera* was associated with inhibition of cholesterol absorption mediated by its fiber content. To test this, fecal cholesterol levels were measured, and an increase in cholesterol excretion was observed in the group fed a high-fat diet supplemented with *N. cochenillifera*. These results suggest that *N. cochenillifera* promotes cholesterol excretion in the feces, thereby contributing to suppression of serum cholesterol levels.

The intestinal barrier plays a critical role in preventing the translocation of pathogens and harmful substances from the intestinal lumen into the systemic circulation. It is maintained by the coordinated function of the mucosal layer, secretory IgA, and tight junction proteins. The mucosal layer is primarily composed of mucin, which is secreted by goblet cells [5]. High-fat-diet intake has been reported to impair intestinal barrier integrity, allowing LPS and other bacterial endotoxins to enter the bloodstream, thus leading to obesity [6]. To assess the effects of *N. cochenillifera* on intestinal immune function, fecal IgA and mucin levels were measured. Cactus supplementation increased fecal IgA levels in both the control and high-fat diet groups. In addition, fecal mucin levels were elevated in response to both dietary conditions following *N. cochenillifera* supplementation. These findings indicate that cactus intake enhances intestinal barrier function by promoting mucin and IgA secretion. Thus, *N. cochenillifera* may exert its anti-obesity effects by strengthening the intestinal barrier, thereby reducing intestinal permeability and subsequent systemic inflammation.

Peyer’s patches, which are part of the gut-associated lymphoid tissue (GALT), play a central role in regulating immune responses to intestinal antigens, including commensal and pathogenic bacteria [30]. Previous studies have reported that cytokine production in Peyer’s patches is associated with obesity-related immune modulation [31]. Therefore, we investigated the mRNA expression levels of obesity-related cytokines in Peyer’s patches to further explore the immunomodulatory effects of *N. cochenillifera*. No significant differences were observed in the gene expression levels of IL-6, which is associated with immune responses and inflammation, or IL-10, which exerts anti-inflammatory effects, among the experimental groups. By contrast, IL-4, which activates B cells and plays a key role in humoral immunity, was significantly upregulated in the group fed a high-fat diet supplemented with *N. cochenillifera*. IL-4 is an anti-inflammatory cytokine that is thought to be involved not only in the activation of anti-inflammatory immune cells but also in adipocyte metabolism. Decreased IL-4 gene expression in adipose tissue and reduced plasma IL-4 levels in obese individuals are associated with increased leptin production, suggesting that IL-4 may play a regulatory role in leptin expression [32]. Furthermore, stimulation of the human colon cancer cell line LS174T with IL-4 alone increased MUC2 mRNA expression by approximately twofold [33], suggesting that cytokines can influence mucin production. However, because LS174T is cancer-derived, it remains unclear how cytokines affect mucin expression in mouse Peyer’s patches. IL-2, which is produced by Th1 cells and involved in cellular immunity, was expressed at lower levels in the FN group supplemented with *N. cochenillifera* than in the corresponding FC group; however, the difference did not reach statistical significance (*p* = 0.0788). This downregulation trend may still be biologically relevant. Previous studies have reported that IL-2 expression in adipose tissue is weakly correlated with body mass index in obese individuals [34], and IL-2 has been implicated in T-cell proliferation and immune regulation [35]. Taken together, these findings suggest that even modest reductions in IL-2 expression could contribute to immunomodulatory actions under high-fat dietary conditions; however, further studies are warranted to confirm this effect. While the present findings suggest that *N. cochenillifera* may modulate immune responses through cytokine regulation, this study has certain limitations. In particular, we did not analyze the expression of genes or proteins involved in key immune signaling pathways in intestinal tissues.

Dysbiosis of the intestinal microbiota is associated with the development of various conditions, including allergies, obesity, and type 2 diabetes. Dietary fiber is beneficial to human health because it acts as a prebiotic that supports the growth of commensal bacteria and contributes to the maintenance of a healthy intestinal environment [36]. We previously reported that *N. cochenillifera* may modulate host immune function by altering the gut microbiota [17]. In the present study, we further examined the effects of *N. cochenillifera* supplementation on the microbial diversity and composition of the gut microbiota by 16SrRNA sequencing. *N. cochenillifera* intake altered β-diversity across the four groups, indicating differences in microbial community structure among individuals, and increased α-diversity, reflecting greater microbial richness within individuals, in the cactus-fed groups. These findings suggest that *N. cochenillifera* supplementation enhances gut microbial diversity.

At the family level, *N. cochenillifera* supplementation increased *Lachnospiraceae* abundance. Telle-Hansen et al. reported that dietary replacement of saturated fatty acids with polyunsaturated fatty acids increased *Lachnospiraceae* abundance and reduced serum cholesterol levels [37]. Although this study did not clarify the precise mechanism by which *Lachnospiraceae* reduces serum total cholesterol, and coprostanol was not measured, our findings suggest a possible link. Changes in the gut microbiota induced by *N. cochenillifera* may affect cholesterol metabolism and its regulation.

Considering the alterations that we observed in gut microbiota composition following *N*. *cochenillifera* supplementation, we measured SCFAs in cecal contents to evaluate the influence of cactus consumption on gut microbial metabolism. In mice fed a normal diet, butyrate levels were significantly increased in the C group; however, no significant differences were observed when compared with the other groups. All analyzed SCFA levels were significantly elevated in the N group compared with the other group. SCFAs produced by gut microbiota, especially butyrate, play crucial roles in lipid metabolism and immune regulation [2,3,32]. Notably, the *Lachnospiraceae* family has been recognized for its ability to produce butyrate and other SCFAs, further emphasizing the significance of this bacterial family in metabolic health. At the genus level, KE159628_g, which belongs to the family *Lachnospiraceae*, showed a pattern similar to that observed at the family level (Figure S1), suggesting that this genus may be a key contributor to the family-level changes.

In a previous study, cecal butyrate levels were decreased in rats fed a high-fat diet, whereas supplementation with purified oligosaccharides such as 1-kestose restored them [38]. The cactus used in this study did not contain purified oligosaccharides, which may explain the absence of a significant butyrate increase in the high-fat diet group. Further studies are needed to isolate and characterize the dietary fiber components of *N. cochenillifera* and assess their individual effects. *N*. *cochenillifera* cladodes contain various bioactive components, such as dietary fiber, polysaccharides, and polyphenols, but the specific constituents responsible for the observed effects were not identified. The contribution of individual components remains unclear. Supplementation with cactus suppressed the increase in *Desulfovibrionaceae* typically observed under high-fat-diet conditions [39], although the specific mechanisms remain unclear. Moreover, this study was limited to 12 weeks, and bile acid and energy metabolism were not analyzed; therefore, long-term effects remain to be clarified. Additionally, the animals were dissected without prior fasting to enable accurate evaluation of fecal weight, fecal markers, and gut microbiota. However, this methodological choice may have influenced serum metabolic parameters and should be considered a limitation. Finally, cage positions within the facility were not rotated; therefore, potential cage effects on gut microbiota and metabolic outcomes cannot be fully excluded.

## 5. Conclusions

The findings from this study suggest that dietary supplementation with *N. cochenillifera* may have a beneficial effect on lipid metabolism; enhance intestinal barrier and immune functions; and modulate gut microbiota, including SCFA production. The observed effects provide mechanistic insights into the potential roles of *N. cochenillifera* in preventing obesity and lifestyle-related diseases. Furthermore, the results from this study support future utilization of *N. cochenillifera* as a sustainable and functional food resource.

## Figures and Tables

**Table 1:** Composition of the test diets (%).

Ingredient	C	CN	FC	FN
Casein	20.0	18.0	19.8	17.8
Cystine	0.3	0.3	0.3	0.3
β-cornstarch	39.7	35.8	―	―
α-cornstarch	13.2	11.9	5.0	4.5
Sucrose	10.0	9.0	33.2	29.8
Maltodextrin	―	―	10.0	9.0
Soybean oil	7.0	6.3	1.0	0.9
Unsalted butter	―	―	20.0	18.0
Cholesterol	―	―	1.0	0.9
Cellulose powder	5.0	4.5	5.0	4.5
Mineral mix (AIN-93G-MX)	3.5	3.2	3.5	3.2
Vitamin mix (AIN-93G-MX)	1.0	0.9	1.0	0.9
Choline bitartrate	0.3	0.2	0.3	0.2
tert-Butylhydroquinone	0.0014	0.0013	0.0042	0.0038
*N. cochenillifera*	―	10.0	―	10.0
Total	100.0	100.0	100.0	100.0

**Table 2:** Feed intake and energy intake for each group.

	C	CN	FC	FN
Food intake (g/day/mouse)	2.75	2.92	2.72	2.81
Energy intake (kcal/day/mouse)	10.1	10.3	11.5	11.4

**Table 3:** Body and tissue weights.

	C	CN	FC	FN
Body weight (g)	31.26 ± 1.28 ^bc^	29.74 ± 1.41 ^c^	35.44 ± 1.30 ^a^	32.55 ± 0.99 ^b^
Liver weight (g)	1.16 ± 0.07 ^c^	1.14 ± 0.04 ^c^	1.86 ± 0.09 ^a^	1.53 ± 0.03 ^b^
Spleen weight (g)	0.09 ± 0.00 ^a^	0.07 ± 0.00 ^b^	0.08 ± 0.00 ^a^	0.08 ± 0.00 ^ab^
Cecum weight (g)	0.26 ± 0.02 ^b^	0.37 ± 0.03 ^a^	0.26 ± 0.02 ^b^	0.27 ± 0.02 ^b^
Cecum content (g)	0.18 ± 0.02 ^b^	0.29 ± 0.06 ^a^	0.19 ± 0.01 ^b^	0.20 ± 0.02 ^b^
Visceral fat (g)	1.69 ± 0.19 ^b^	1.53 ± 0.22 ^b^	2.86 ± 0.13 ^a^	2.42 ± 0.09 ^a^
Triceps muscle (g)	0.21 ± 0.03 ^a^	0.20 ± 0.02 ^a^	0.19 ± 0.01 ^a^	0.18 ± 0.01 ^a^

Data are expressed as the mean ± SEM (*n* = 10). Means for variables without a common letter differ significantly (*p* < 0.05).

## Data Availability

Not applicable. The original contributions presented in the study are included in the article, further inquiries can be directed to the corresponding author.

## Supplementary Materials

The following supporting information can be downloaded at https://www.mdpi.com/article/10.3390/nu17172844/s1, Table S1: PCR primers used in the present study. References are cited in the Supplementary Materials. Figure S1: LDA effect size (LEfSe) analysis of cecal microbiota at the genus level.

## Abbreviations

Ig	Immunoglobulin
SCFAs	Short-chain fatty acids
Th1	T-helper type 1
LPS	Lipopolysaccharide
FAO	Food and Agriculture Organization
F2WTD	Fructose 20% Western-Type Diet
C	Control diet groups
CN	Control diet + 10% *N*. *cochenillifera* groups
FC	F2WTD diet groups
FN	F2WTD + 10% *N*. *cochenillifera* diet groups
TG	Triglycerides
FFAs	Free fatty acids
AST	Aspartate aminotransferase
ALT	Alanine aminotransferase
ELISA	Enzyme-linked immunosorbent assay
RTU	Ready-to-use
IL	Interleukin
