# Supplementary material for: Modulation of the Gut Microbiota by Nopalea cochenillifera (Prickly Pear Cactus) Contributes to Improved Lipid Metabolism and Immune Function
Source: Nutrients. 2025 Aug 31;17(17):2844. doi: 10.3390/nu17172844 (PMC12429981; doi:10.3390/nu17172844)
Supplement: Supplementary file 1 [file nutrients-17-02844-s001.zip › Figure S1 title;LDA effect size (LEfSe) analysis of cecal microbiota at the genus level..pdf]

Supplementary Figure S1. Linear discriminant analysis (LDA) effect size was used to identify microbial taxa significantly associated with both cactus-fed groups compared with their respec-tive control groups at the genus level within the cecal microbiota. (A) Comparison between groups C and N. (B) Comparison between groups FC and FN. Thresholds for significance were set at p<0.05 and |LDA effect size| > 4.0. Blue bars indicate the magnitude of the LDA effect size, whereas red bars represent relative taxonomic abundance. The *KE159628_g, LT706945_g, KE159810_g, PAC002482_g, PAC002448_g*, and *PAC000664_g* are members of the Lachnospiraceae, Desulfovibrionaceae, Lachnospiraceae, order Bacteroidales, Muribaculaceae, and Lachnospiraceae, respectively.

(A)

Taxon name	*P*-value	*Q*-value	LDA effect size	Average relative abundance (%)	
				C	N
Faecalibaculum	0.01911	0.0425	4.70177	21.24224	10.46376
KE159628_g	0.00033	0.0018	4.55453	0.00046	7.41944
Mucispirillum	0.0015	0.00611	4.45549	8.01359	2.3051
LT706945_g	0.00815	0.02104	4.41075	11.63434	7.08014
Turicibacter	0.00194	0.00669	4.30631	0.66763	4.77139
KE159810_g	0.00407	0.01216	4.17875	7.29281	4.18007
PAC002482_g	0.00016	0.0014	4.11104	2.96884	0.38176
PAC002448_g	0.01556	0.03564	4.09472	0.74967	2.90955
Ruminococcus	0.00005	0.0014	4.05179	0	2.31671

(B)

Taxon name	*P*-value	*Q*-value	LDA effect size	Average relative abundance (%)	
				FC	FN
KE159628_g	0.00011	0.00107	4.86923	0.00729	15.09939
Bacteroides	0.00051	0.00242	4.73135	19.3318	7.74634
LT706945_g	0.00016	0.00107	4.67428	19.23646	9.20779
Faecalibaculum	0.01017	0.02893	4.4022	9.68294	6.43183
Alloprevotella	0.00021	0.00119	4.32774	5.10549	0.96414
Parabacteroides	0.00016	0.00107	4.24768	4.18473	0.58845
Clostridium_g21	0.0025	0.00848	4.22104	5.24753	8.81387
Eubacterium_g6	0.00067	0.00276	4.04354	0.61469	2.71515
Mucispirillum	0.01261	0.03424	4.02458	5.20729	2.73526
PAC000664_g	0.00088	0.00351	4.01738	3.56676	1.59989
