# Supplementary material for: Modulation of the Gut Microbiota by Nopalea cochenillifera (Prickly Pear Cactus) Contributes to Improved Lipid Metabolism and Immune Function
Source: Nutrients. 2025 Aug 31;17(17):2844. doi: 10.3390/nu17172844 (PMC12429981; doi:10.3390/nu17172844)
Supplement: Supplementary file 1 [file nutrients-17-02844-s001.zip › Table S1 title; PCR primers used in the present study..pdf]

Table S1. PCR primers used in the present study

target gene	Primer name	Oligonucleotide sequence	Refarence	Used for
18S rRNA	18S rRNA forward	TTGGAGGGCAAGTCTGGTG	[40]	RT-qPCR
	18S rRNA reverse	CCGCTCCCAAGATCCAACTA	[40]	
IL-2	IL-2 forward	AACCTGAAACTCCCCAGGAT	[41]	RT-qPCR
	IL-2 reverse	CGCAGAGGTCCAAGTTCATC	[41]	
IL-4	IL-4 forward	GGCATTTTGAACGAGGTCAC	[41]	RT-qPCR
	IL-4 reverse	AAATATGCGAAGCACCTTGG	[41]	
IL-6	IL-6 forward	AACGATGATGCACTTGCAGA	[41]	RT-qPCR
	IL-6 reverse	GGTACTCCAGAAGACCAGAGGA	[41]	
IL-10	IL-10 forward	TGAATTCCCTGGGTGAGAAG	[41]	RT-qPCR
	IL-10 reverse	TGGCCTTGTAGACACCTTGG	[41]	

Lower case sequences indicate 15 bases homologous to the entry vector.

[40] Olmeda D, Cerezo-Wallis D, Mucientes C, Calvo TG, Cañón E, Alonso-Curbelo D, et al. Live imaging of neolymphangiogenesis identifies acute antimetastatic roles of dsRNA mimics. EMBO Mol Med. 2021;13:e12924; doi:10.15252/emmm.202012924.

[41] Banerjee ER, Henderson WR. Role of T cells in a gp91phox knockout murine model of acute allergic asthma. Allergy Asthma Clin Immunol. 2013;9:6; doi:10.1186/1710-1492-9-6.
